# In vivo assessment of a single adenine mutation in 5′UTR of Endothelin-1 gene in paediatric cases with severe pulmonary hypertension: an observational study

**DOI:** 10.1186/s13104-021-05609-5

**Published:** 2021-05-19

**Authors:** Abhishek Kumar, Minati Choudhury, Sakshi Dhingra Batra, Kriti Sikri, Anushree Gupta

**Affiliations:** 1 Department of Biotechnology, All India Institute of Medical Sciences, New Delhi, India; 2 Department of Cardiac Anaesthesia, All India Institute of Medical Sciences, New Delhi, India

**Keywords:** Endothelin-1, Severe pulmonary hypertension, Congenital heart disease, Cyanotic, Acyanotic, Single nucleotide polymorphisms, 5′Untranslated region (5′UTR), Upstream regulatory element, Haplotype, Allele

## Abstract

**Objective:**

Endothelin-1 plays an important role in the pathogenesis of severe pulmonary hypertension. The + 139 ‘A’, adenine insertion variant in 5′UTR of *edn1* gene has been reported to be associated with increased expression of Endothelin-1 in vitro. The aim of present study was to explore the association of this variant with the circulating levels of Endothelin-1 in vivo using archived DNA and plasma samples from 38 paediatric congenital heart disease (cyanotic and acyanotic) patients with severe pulmonary hypertension.

**Results:**

The plasma Endothelin-1 levels were highly varied ranging from 1.63 to75.16 pg/ml. The + 139 ‘A’ insertion variant in 5′UTR of *edn1* was seen in 8 out of 38 cases with only one acyanotic sample demonstrating homozygosity of inserted ‘A’ allele at + 139 site (4A/4A genotype). The plasma Endothelin-1 levels in children with homozygous variant 3A/3A genotype were comparable in cyanotic and acyanotic groups. Lone 4A/4A acyanotic sample had ET-1 levels similar to the median value of ET-1 associated with 3A/3A genotype and was absent in cyanotic group presumably due to deleterious higher ET-1 levels. The discussed observations, limited by the small sample size, are suggestive of homozygous adenine insertion variant posing a risk in cyanotic babies with Severe Pulmonary Hypertension.

**Supplementary Information:**

The online version contains supplementary material available at 10.1186/s13104-021-05609-5.

## Introduction

Severe pulmonary hypertension (SPH) is a rare disease with an incidence of 2**–**3 per million and a prevalence of 25**–**50 per million [1]. Recent developments have resulted in improved prognosis, however, mortality is still high in the pediatric population [2–5]. Patients with congenital heart defects (CHD) often reveal pulmonary hypertension associated with progressive vascular changes leading to pulmonary vascular disease (PVD). Although the etiology of PVD is multifactorial, Endothelin-1 (ET-1), a potent vasoconstrictive peptide, has been implicated in the pathogenesis of pulmonary hypertension (PH) and the Endothelin-1 pathway is an important target in PH-specific drug therapy [5]. ET-1 is produced and secreted into circulation by the endothelial cells of the pulmonary vessels. Several stimuli lead to increase in synthesis of ET-1 in pulmonary hypertension [6–8]

**Fig. 1:**
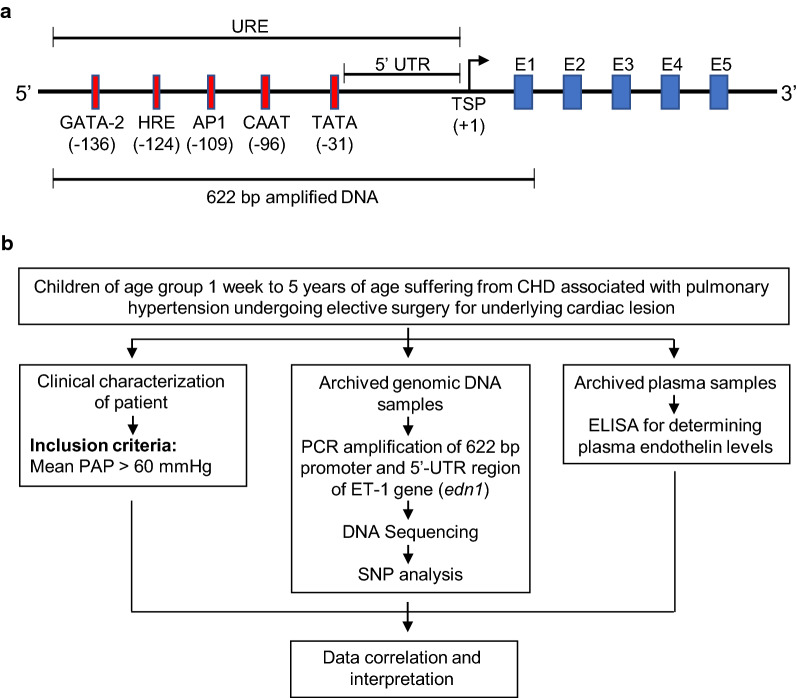
Study outline. **A** Schematic representation of the ET-1 gene showing 5′ upstream regulatory elements (5′URE) and the five exons of *edn1* gene represented by boxes E1 to E5; the amplified region of 622 bps (−288 to + 344 position) including proximal promoter region, 5′ UTR and exon 1 as shown were amplified using primers—forward primer: AGCTTGCAAAGGGGAAGC; reverse primer: ACCTGTTTCTGGAGCTCC) using *edn1* gene reference sequence (locus NG_016196 on chromosome 6) available at NCBI [17]. **B** Outline of the study depicting the three parallel arms undertaken, namely, characterization of clinical samples (parallel study), gene sequencing and measurement of ET-1 levels by ELISA

The ET-1 gene (*edn1*) consists of five exons distributed over 6836 bps of the genome located on chromosome 6 (6p23–p24) [9].The large precursor protein, preproendothelin-1, comprised of 212 amino acids, encoded by 2026 nucleotide mRNA (excluding the poly-A tail), is processed to the mature 21 aa biologically active peptide in several steps [10, 11]. It is likely that regulatory mechanisms exist for each of the post-translational processing steps; however, the general prevailing scientific consensus is that transcription is the primary mechanism of ET-1 regulation [12–14]. The arrangement of ET-1 gene regulatory region comprising of the proximal promoter elements and 5′UTR, upstream of the 5 exons is represented schematically in Fig. 1A.

Alterations in ET-1 expression patterns have been reported in the pathogenesis and progression of various disease states that also include congenital cyanotic heart disease and pulmonary hypertension where there is an absolute increase in ET-1 levels across pulmonary circulation compared with non-pulmonary hypertension group [15]. Studies have demonstrated alteration in ET-1 signalling in new-borns, infants and children with congenital heart disease that are associated with pulmonary blood flow [16].

In the present work, the minimal promoter elements of ET-1 gene and its 5′UTR have been examined in children with CHD and suffering from SPH. This study relates the role of regulatory sequences on the variations in plasma Endothelin-1 levels which seems to affect the severity of the disease.

## Main text

### Methods

#### Samples and study design

Archived frozen DNA and plasma samples isolated from peripheral blood of unrelated congenital heart disease children (both cyanotic and acyanotic) between 1 week–5 years of age (sample size = 38) with severe pulmonary hypertension formed the starting material of the study. The children with mean pulmonary arterial pressure (PAP) > 60 mm Hg (as measured in the cardiac CATH lab during diagnostic procedure) were undergoing elective surgery to repair cardiac defects at Cardio-thoracic and Neurosciences Centre AIIMS, New Delhi, India (Additional file 1: Table S1). The promoter and 5′-UTR of *edn1* gene from the DNA samples were PCR amplified and sequenced and the serum samples were used for measurement of Endothelin-1 levels (Fig. 1).

#### Screening for mutation in the promoter and 5′UTR region of the ET-1 gene by PCR and sequencing

The 622 bps region including proximal promoter, 5′UTR and exon-1 of Endothelin-1 gene were PCR amplified using designed primers (Fig. 1A). The amplified products were purified by Qiaquick direct PCR product purification kit (Qiagen, USA) and sequenced by using ABI PRISM Big Dye Terminator Method (kit version 3.1, PE Applied Biosystem, CA, USA). Sequencing chromatograms obtained for all 38 PCR products were analysed for single nucleotide polymorphisms (SNPs) by aligning proximal promoter and 5′UTR sequences from both cyanotic and acyanotic samples with the reference sequence (NG_016196.1) of *edn1 *gene using BioEdit sequence alignment editor [18]**.**

#### Enzyme immuno-assay for estimation of plasma ET-1 levels

Levels of ET-1 in the plasma samples were determined by an acetylcholinesterase-based immunoassay- ACTEM (Cayman Chemical Co. U.S.A. #583,151), a double-antibody ‘sandwich’ ELISA kit, according to the manufacturer’s instructions.

#### Statistical methods

Wherever applicable the statistical significance was calculated using non-parametric Mann–Whitney test.

### Results

#### The promoter elements of edn1 gene are strongly conserved

A careful analysis of the aligned sequences revealed strong conservation of all the 4 promoter elements (GATA-2, HRE, AP-1 and CAAT Box) in both cyanotic and acyanotic groups. Further, the spacing between the various promoter elements was also maintained (Additional file 2: Figure S1). The clustering and strong conservation of promoter elements, all contained within 40 bps (−136 to −96), is indicative of a tight regulation of *edn1* gene expression at the transcriptional level.

#### Occurrence of adenine at + 139 position in ﻿5’UTR of edn1 ﻿﻿gene

**Fig. 2:**
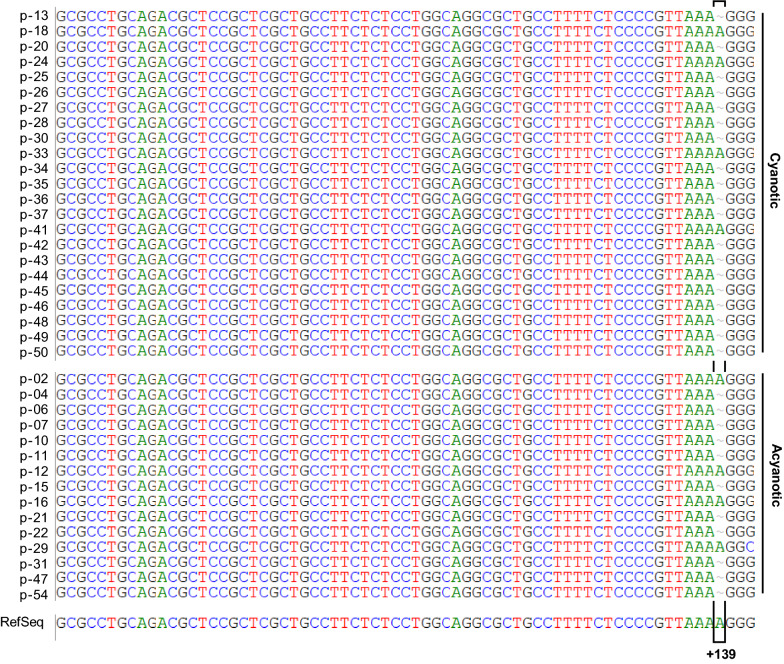
Alignment of 5′-UTR region of *edn1* gene from patient samples. The amplified 5′ UTR regions from *edn1* gene from 38 patient samples in the cyanotic and acyanotic groups are shown aligned to the reference sequence (NG_016196.1). The In/del of Adenine (A) at position + 139 from the transcription initiation site is depicted

The *edn1* gene contains a 5′UTR which is 268 bps long and stretches from transcription start site (+ 1) to the translation start codon ATG (+ 269). The alignment of 5′UTR of sequences of all samples with the 5′UTR of RefSeq of *edn1*(NG_016196.1) revealed that 30 out of 38 sequences showed a deletion of ‘A’ at + 139 position of *edn1* irrespective of cyanosis or acyanosis. In other words, these sequences had ‘AAA’ (3A) at positions + 136, + 137 and + 138 followed by a ‘G’ at + 139 position whereas the reference sequence has another ‘A’ at position + 139 resulting in a 4A genotype followed by a ‘G’ at + 140 position (Fig. 2). Among the eight cases which are without deletion for ‘A’ at + 139, seven cases have a heterozygous genotype (3A/4A) as revealed by double peaks of ‘A’ and ‘G’ of equal intensity and height in the sequencing chromatogram and only one case shows homozygous 4A/4A condition at this site (Additional file 2: Figure S2).

The frequency distribution of different allele types in the 38 samples revealed 78.95% (30/38) samples to be homozygous for 3A/3A, 18.42% (7/38) were heterozygous for 3A/4A and 2.63% (1/38) were homozygous for 4A/4A. The occurrence of 3A/3A allele was higher in the cyanotic group (66.6%) as compared to acyanotic group (33.3%), while the occurrence of heterozygous 3A/4A allele type was 43% in the cyanotic and 57% in acyanotic groups. The homozygous 4A/4A allele was rare, being completely absent in the cyanotic group and only 1 sample in acyanotic group (Additional file 1: Table S2).

#### Variation in plasma ET-1 levels in children with severe pulmonary hypertension

**Fig. 3:**
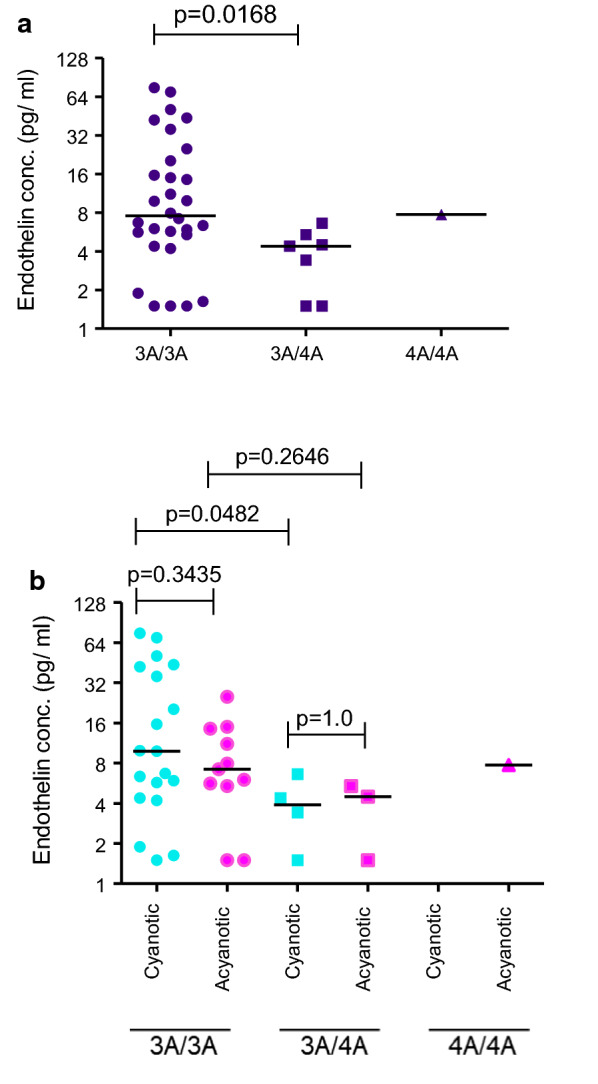
Association of plasma Endothelin-1 levels with different alleles at + 139 position in 5′UTR of *edn1* gene.The distribution of ET-1 levels in (**a**) CHD group and (**b**) Cyanotic (cyan) and acyanotic (pink) groups is represented for each patient by filled circles (3A/3A), squares (3A/4A) and triangles (4A/4A) and the median value for each group is represented by a horizontal line. [*p-value calculated using non-parametric Mann–Whitney test]

A wide variation in plasma ET-levels was observed in both cyanotic and acyanotic children with a minimum value of 1.63 pg/ml and a maximum value of 75.16 pg/ml with a median value of 6.495 pg/ml (Fig. 3A and Additional file 1: Table S1). However, the median levels of plasma Endothelin-1 in cyanotic (6.670 pg/ml) and acyanotic (5.690 pg/ml) groups were statistically insignificant (p-value = 0.2492) **(**Additional file 2: Figure S3).

#### Association of + 139 position adenine (A) In/del with plasma ET-1 level

The distribution pattern of plasma ET-1 level in different allele types, 3A/3A homozygous, 3A/4A heterozygous and 4A/4A homozygous was compared in the CHD group as a whole and within the cyanotic and acyanotic sub-groups. A significant difference (p-value = 0.0168) in plasma ET-1 levels was found between the allelic group 3A/3A homozygous and 3A/4A heterozygous in the study group of CHD cases (Fig. 3a), this difference being marginally more significant (p-value = 0.0482) in cyanotic than in acyanotic group (p-value = 0.2646; Fig. 3b). Within the allelic groups 3A/3A and 3A/4A there was no difference in the plasma ET-1 levels between cyanotic and acyanotic groups. Among 38 cases, only one subject belonging to acyanotic group displayed 4A/4A homozygous allelic condition. Hence, statistical significance could not be applied to this group. The median levels of ET-1 in all groups of tested samples are high when compared to reported plasma ET-1 levels of 1.59 ± 0.32 pg/ml [19] in healthy subjects.

### Discussion

Our pilot study demonstrates for the first time the significance of a single minor allele adenine insertion variant in 5′UTR of *edn1* gene in paediatric cases of congenital heart disease with severe pulmonary hypertension. The study adds important insights into *edn1* genotype–phenotype associations in vivo that are at variance with earlier in vitro studies primarily due to the extremely low frequency of occurrence or complete absence of 4A/4A genotype in diseased condition. The results highlight the potential role of 5′UTR of *edn-1* gene in modulating the expression of ET-1 in CHD patients under study, by unknown mechanisms, which in turn may alter the course of congenital heart anomaly after it has been initiated. Significantly higher frequency of occurrence of 3A/3A genotype in cyanotic group, similar frequency of occurrence of 3A/4A genotype in cyanotic and acyanotic groups and complete absence of 4A/4A genotype in cyanotic cases are together suggestive of the 4A/4A genotype being risky in cyanotic group.

The regulation of ET-1 levels is directly rooted at the level of transcription, as supported by previous studies [20]. A well conserved proximal promoter, while indicative of overall functional importance of *edn1* gene, cannot explain the occurrence of varied circulating levels of Endothelin-1 as observed in this study. Since, non-coding elements in 5′UTR have been reported to affect promoter activity [21–24] via binding of sequence-specific mRNA-binding proteins, this regulatory region was expected to modulate the expression of mature ET-1 and was examined further.The sequence of entire 5′UTR of *edn-1* gene including the occurrence of single + 139 in/del adenine mutation, along with the associated circulating plasma ET-1 levels were analysed in all samples.

Both 3A/3A and 4A/4A genotypes showed similar median values for immune-reactive plasma ET-1 levels (7.57 pg/ml and 7.75 pg/ml respectively. An earlier in vitro study involving transfection experiments with endothelial cell cultures showed that the homozygous 4A/4A genotype is associated with significantly higher levels of ET-1 when compared to either 3A/3A or 3A/4A genotypes where the expression levels were comparable [25]. The present study showed association of relatively lower plasma ET-1 levels (4.39 pg/ml) with the heterozygous 3A/4A genotype compared to the 3A/3A genotype. However, studies with adult samples of CAD showed similar levels of ET-1 with 3A/3A or 3A/4A genotypes (our unpublished observations) and even higher levels of ET-1 with 4A/4A genotype in consonance with the in vitro study [25]. The difference in genotypic association of ET-1 levels in the present study lies in the choice of the tested population namely that of babies with cardiac lesions. The high levels of endothelin-1 expression associated with 4A/4A genotype are probably too high to be tolerated under the cyanotic condition owing to the established deleterious role of endothelin-1 in increasing pulmonary arterial pressure. Even in acyanotic condition, the 4A/4A associated expression levels of ET-1 are tolerated if they are comparable to threshold levels associated with 3A/3A and 3A/4A genotypes. Hence, It may be speculated that there is an association of even higher plasma ET-1 levels in 4A/4A cyanotic genotype which cannot be scored owing to the high severity of the disease, possibly leading to loss of life and hence the complete absence of 4A/4A genotype in this group. This raises the question “*is the minor 4A allele a risk allele in cyanotic babies*?’ There is a selection pressure acting against the 4A/4A genotype presumably due to the associated very high levels of ET-1. Large-scale genome wide association studies (GWAS) by computational methods have postulated enrichment of risk alleles in minor alleles, especially for variants with low minor allele frequencies [26]. Our findings provide an evidence for the minor allele becoming a risk allele in cyanotic cases, a finding that needs to be explored further. A more detailed and careful follow up of CHD + SPH cases with the 4A haplotype with appropriate controls may be required to probe into the clinical significance and its associated implications on genetic testing and counselling, of this finding that in turn will shape future research to be pursued as well as appropriate interventions in therapy and management.

## Limitations

This was an exploratory study carried out on a small group of paediatric cases (n = 38) suffering from congenital heart defects complicated with SPH and undergoing corrective surgery. Since the samples for the study were obtained from CHD surgical clinics, controls with SPH alone and healthy age-matched controls could not be obtained for comparison, hence, our observations are speculative in nature. We could not apply statistical tests of significance to the 4A/4A genotypic group because of the small sample size of children and the rarity of occurrence of 4A haplotype.

## Supplementary Information

**Additional file1**: ** Table S1.** Baseline Clinical characteristics of CHD patients with severe pulmonary hypertension from whom the Blood Samples used in the study were derived. **Table S2.** Distribution of different allele types at +139 position of ET-1 gene in cyanotic and acyanotic groups.

**Additional file2**: **Figure S1.** Alignment of promoter region of edn1 gene from patient samples. The amplified promoter regions from edn1 gene depicting the conserved promoter elements from 38 patient samples in the cyanotic and acyanotic groups are shown aligned to the reference sequence (NG_016196.1). **Figure S2.** Sequencing chromatogram of the 5’ UTR of the ET-1 gene showing 3A/3A homozygous, 4A/4A homozygous and 3A/4A heterozygous allelic condition with an insertion of adenine (A) at +139 position from the transcription initiation site. **Figure S3.** Plasma Endothelin-1 (ET-1) levels (pg/ml) in CHD patients. The distribution of ET-1 levels in both Cyanotic and Acyanotic groups is represented for each patient with the median value for each group. [*p-value calculated using non-parametric Mann-Whitney test].

## Data Availability

The datasets used and/or analyzed during the current study are available from the corresponding author on reasonable request.

## Abbreviations

ET-1: Endothelin-1
SPH: Severe pulmonary hypertension
CHD: Congenital heart disease
SNPs: Single nucleotide polymorphisms
5′UTR: 5′Untranslated region
URE: Upstream regulatory element
GWAS: Genome wide association studies
In/del: Insertion/deletion
