# Supplementary material for: In vivo assessment of a single adenine mutation in 5′UTR of Endothelin-1 gene in paediatric cases with severe pulmonary hypertension: an observational study
Source: BMC Res Notes. 2021 May 19;14:194. doi: 10.1186/s13104-021-05609-5 (PMC8136217; doi:10.1186/s13104-021-05609-5)
Supplement: Supplementary file 1 — Additional file1: Table S1. Baseline Clinical characteristics of CHD patients with severe pulmonary hypertension from whom the Blood Samples used in the study were derived. Table S2. Distribution of different allele types at +139 position of ET-1 gene in cyanotic and acyanotic groups. [file 13104_2021_5609_MOESM1_ESM.doc]

**Additional File 1**

**Table S1** Baseline Clinical characteristics of CHD patients with severe pulmonary hypertension from whom the Blood Samples used in the study were derived.

**Cyanotic group**

| **Patient code** | **Age (months)** | **ET-1 conc (pg/ ml)** | **Diagnosis** |
| --- | --- | --- | --- |
| **P13** | 7 | 1.63 | Truncus Arteriosus (Type A) |
| **P18** | 48 | 6.63 | Patent LMBTS, confluent Pas VSD, PS, TGA |
| **P20** | 7 | 9.93 | Supracardiac TAPVC |
| **P24** | 24 | 1.63 | Truncus Arteriosus (Type I) |
| **P25** | 2.3 | 4.39 | dTGA VSD |
| **P26** | 6 | 6.36 | Truncus arteriosus type-1 |
| **P27** | 12 | 6.71 | TAPVC, supracardiac type |
| **P28** | 8 | 5.73 | Truncus arteriosus type-1, Interrupted Aortic arch |
| **P30** | 30 | 4.21 | Persistent Truncus Arteriosus Type 1 |
| **P33** | 6 | 4.39 | TAPVC, unobstructed |
| **P34** | 3 | 15.64 | Supracardiac TAPVC, mixed type (Obstructive) |
| **P35** | 1 | 1.63 | Hemi truncus |
| **P36** | 60 | 1.89 | Large P/MVSD |
| **P37** | 7 | 9.84 | TAPVC (Mixed cardiac/supracardiac, unobstructed) |
| **P41** | 3.6 | 3.41 | P/TAPVC Repair (side to side anastomosis of LA to Vertical Vein) ASD |
| **P42** | 4 | 35.7 | Supra cardiac TAPVC, restrictive ASD, NSR, No CHF |
| **P43** | 1.5 | 69.78 | Supra cardiac TAPVC |
| **P44** | 0.5 | 20.33 | CHD with SPH |
| **P45** | 12 | 43.67 | Supracardiac TAPVC, unrestrictive ASD |
| **P46** | 2 | 5.91 | Infracardiac TAPVC - obstructed, large ASD |
| **P48** | 24 | 50.89 | CHD with SPH |
| **P49** | 17 | 75.16 | CHD with SPH |
| **P50** | 3 | 42.19 | Patent truncus arteriosus, small, ASD |

**Acyanotic group**

| **Patient code** | **Age (months)** | **ET-1 conc (pg/ ml)** | **Diagnosis** |
| --- | --- | --- | --- |
| **P2** | 2 | 7.75 | VSD, AP window, PA, ASD |
| **P4** | 9 | 7.93 | Truncus arteriosus (Type I aortic arch interrupted, VSD) |
| **P6** | 8 | 5.38 | Complete AVSD |
| **P7** | 6 | 7.21 | CHD with SPH |
| **P10** | 7 | 1.63 | CHD with SPH |
| **P11** | 36 | 11.14 | Complete AVSD |
| **P12** | 11 | 1.63 | large PM VSD |
| **P15** | 2 | 1.63 | Persistent Truncus Arteriosus (Type I) |
| **P16** | 4 | 4.5 | P/Repair, Interrupted aortic arch (Type A) |
| **P21** | 30 | 6 | Large PM VSD |
| **P22** | 36 | 14.5 | Large subpulmonic VSD |
| **P29** | 18 | 5.38 | AVSD, Intermediate type |
| **P31** | 1 | 5.64 | TAPVC, large ASD |
| **P47** | 8 | 25.15 | AVSD Repair + TV repair |
| **P54** | 12 | 14.96 | VSD |

**Note:**

PA-VSD, Pulmonary Atresia with ventricular septal defect; PS, Pulmonary Stenosis; VSD, Ventricular Septal Defect; TGA, Transposition of the Great Arteries; dTGA, dextro TGA; TAPVC, Total Anomalous Pulmonary Venous Connection; P/MVSD, peri-membranous ventricular septal defect; LA, Left Atrium; ASD, Atrial Septum Defect; CHD, Congenital Heart Disease; SPH, severe pulmonary hypertension; AP Window, Aortopulmonary Window; TV-Repair, Tricuspid Valve Repair.

**Table S2.** Distribution of different allele types at +139 position of ET-1 gene in cyanotic and acyanotic groups.

| Genotype | Cyanotic | Acyanotic |
| --- | --- | --- |
| 3A/3A | 20 (66.6%) | 11 (33.3%) |
| 3A/4A | 4 (57%) | 3 (43%) |
| 4A/4A |  | 1 (100%) |

* Fisher’s exact test p-value = 0.678.
