# Supplementary material for: In vivo assessment of a single adenine mutation in 5′UTR of Endothelin-1 gene in paediatric cases with severe pulmonary hypertension: an observational study
Source: BMC Res Notes. 2021 May 19;14:194. doi: 10.1186/s13104-021-05609-5 (PMC8136217; doi:10.1186/s13104-021-05609-5)
Supplement: Supplementary file 2 — Additional file2: Figure S1. Alignment of promoter region of edn1 gene from patient samples. The amplified promoter regions from edn1 gene depicting the conserved promoter elements from 38 patient samples in the cyanotic and acyanotic groups are shown aligned to the reference sequence (NG_016196.1). Figure S2. Sequencing chromatogram of the 5’ UTR of the ET-1 gene showing 3A/3A homozygous, 4A/4A homozygous and 3A/4A heterozygous allelic condition with an insertion of adenine (A) at +139 position from the transcription initiation site. Figure S3. Plasma Endothelin-1 (ET-1) levels (pg/ml) in CHD patients. The distribution of ET-1 levels in both Cyanotic and Acyanotic groups is represented for each patient with the median value for each group. [*p-value calculated using non-parametric Mann-Whitney test]. [file 13104_2021_5609_MOESM2_ESM.doc]

**Additional File 2**

**
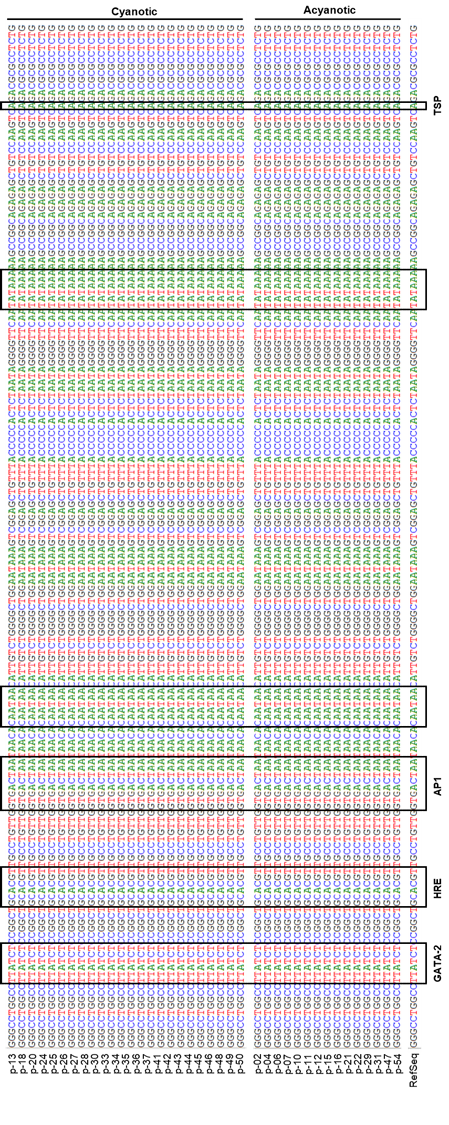
**

**Figure S1: Alignment of promoter region of edn1 gene from patient samples.** The amplified promoter regions from edn1 gene depicting the conserved promoter elements from 38 patient samples in the cyanotic and acyanotic groups are shown aligned to the reference sequence (NG_016196.1).

**
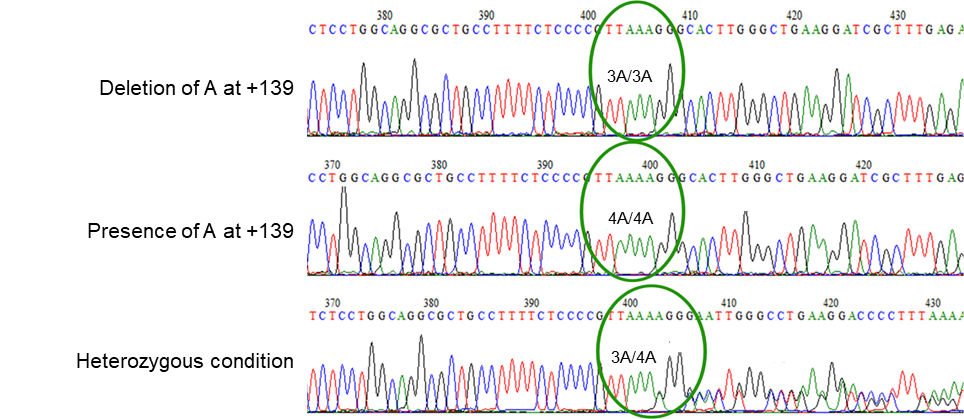
**

**Figure S2:** Sequencing chromatogram of the 5’ UTR of the ET-1 gene showing 3A/3A homozygous, 4A/4A homozygous and 3A/4A heterozygous allelic condition with an insertion of adenine (A) at +139 position from the transcription initiation site.

**
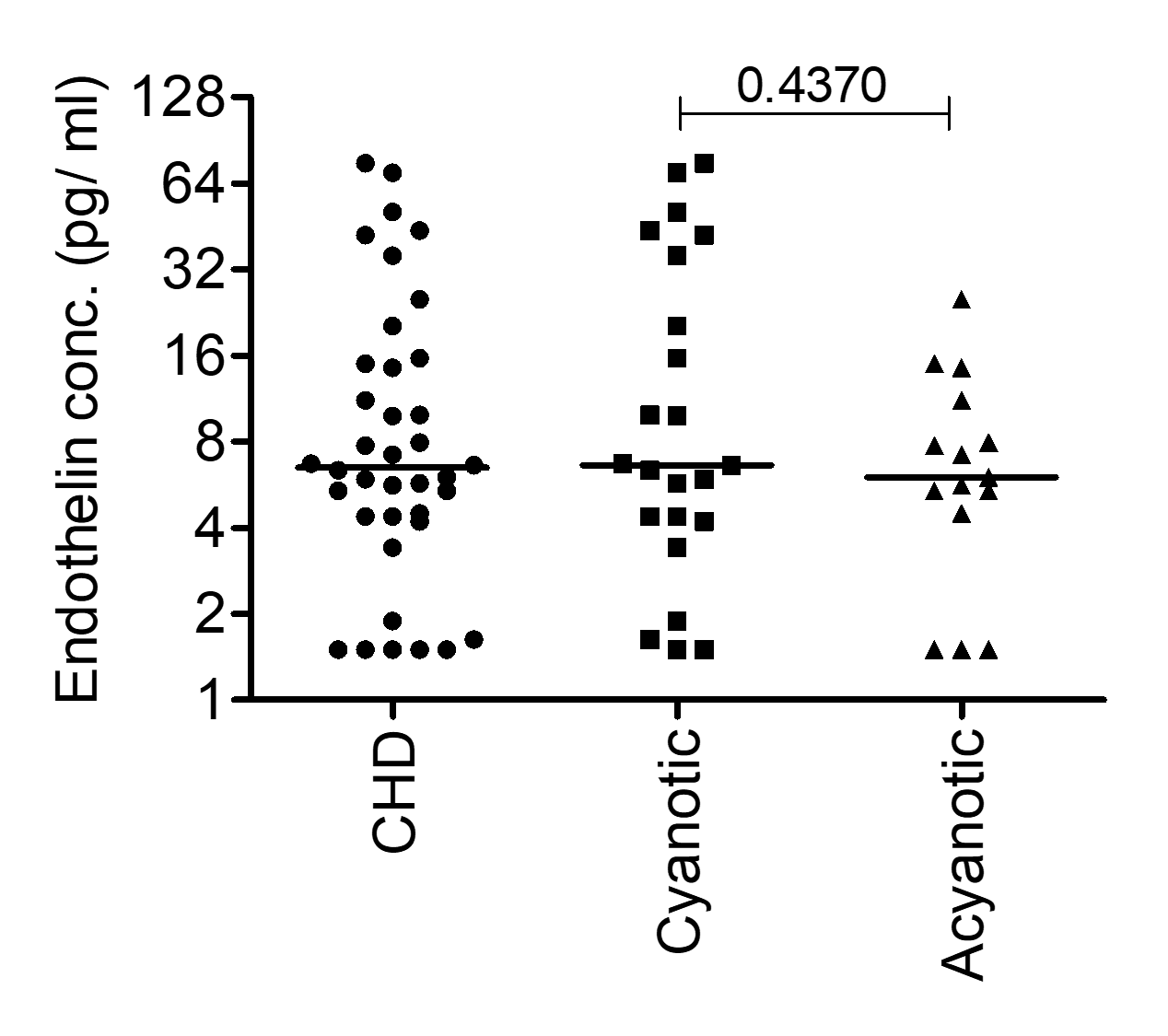
**

**Figure S3: Plasma Endothelin-1 (ET-1) levels (pg/ml) in CHD patients.** The distribution of ET-1 levels in both Cyanotic and Acyanotic groups is represented for each patient with the median value for each group. [*p-value calculated using non-parametric Mann-Whitney test].
